# Socioeconomic equality in initiation of biologic treatment in Danish patients with inflammatory bowel disease

**DOI:** 10.1007/s00508-024-02376-8

**Published:** 2024-05-24

**Authors:** Sofie Ronja Petersen, Nathalie Fogh Rasmussen, Agnete Overgaard Donskov, Lau Caspar Thygesen, Kim Rose Olsen, Linda Juel Ahrenfeldt, Vibeke Andersen

**Affiliations:** 1 Molecular Diagnostic and Clinical Research Unit, University Hospital of Southern Denmark, Kresten Phillipsens vej 15, Aabenraa, Denmark; 2 Institute of Regional Health Research, University of Southern Denmark, Odense, Denmark; 3 Hospital Pharmacy Research Unit, University Hospital of Southern Denmark, Aabenraa, Denmark; 4 Department of Geriatrics, Hospital of Southern Denmark, Aabenraa, Denmark; 5 National Institute of Public Health, University of Southern Denmark, Copenhagen, Denmark; 6 Danish Centre for Health Economics—DaCHE, University of Southern Denmark, Odense, Denmark; 7 Unit for Epidemiology, Biostatistics and Biodemography, Department of Public Health, University of Southern Denmark, Odense, Denmark

**Keywords:** Inflammatory Bowel Disease, Time to treatment, Education, Income, Occupation

## Abstract

**Background:**

Low socioeconomic status is associated with disadvantages in health outcomes and delivery of medical care in patients with Inflammatory Bowel Disease (IBD). Inequality in the utilisation of biologic treatment is largely unexplored.

**Aim:**

To explore the potential association of socioeconomic status and time to first biologic treatment in a population-based IBD cohort.

**Methods:**

All 37,380 IBD incidences between 2000 and 2017 from the Danish National Patient Register were identified and linked to socioeconomic information including educational level, income and occupational status at diagnosis. Hazard ratios for receiving biologic treatment among socioeconomic groups were estimated using Cox proportional hazard regression.

**Results:**

No difference in time between diagnosis and biologic treatment initiation was found comparing patients with upper secondary, vocational, or academic education to those with lower secondary education in patients with IBD. Patients with Crohn’s disease in the two highest income quartiles received biologic treatment earlier (HR 1.16; 95% CI: 1.04; 1.30 & HR 1.15; 95% CI: 1.03; 1.30). An elevated treatment rate was found for persons with “other” occupational status (unspecified source of income) compared to employed persons in patients with ulcerative colitis (HR 1.36; 95% CI: 1.11; 1.66), but not in patients with Crohn’s disease.

**Conclusion:**

This study revealed equal initiation of biologic treatment among patients with IBD across different educational background, income and occupational status. However, results are limited to a setting with free universal healthcare coverage and treatment needs should be considered and addressed in future research.

**Supplementary Information:**

The online version of this article (10.1007/s00508-024-02376-8) contains supplementary material, which is available to authorized users.

## Introduction

### Background and rationale

Inflammatory bowel diseases (IBD) comprise ulcerative colitis (UC) and Crohn’s disease (CD) and are characterised by an overreactive immune response to normal conditions in the digestive system [1, 2]. IBDs represent an increasing public health burden in high-income and newly industrialised countries [3]. The diseases cause strong economic and social impacts, as the diagnosis is often issued at the economically most productive age and related to disability and compromised life quality [4]. In this context, a well-tailored long-term disease management is crucial to ensure the highest possible quality of life. This frequently includes the onset of biologic treatment [5]. Whilst the aetiology of CD and UC is complex and not yet fully understood, positive socioeconomic and geographic gradients in IBD prevalence and treatment choice have been detected [3]. This was shown on the population level, however, evidence on the relevance of individual socioeconomic status (SES) in disease management is lacking.

Despite publicly financed health care, social inequality is still a concern regarding health-related outcomes and service use [6]. Therefore, the meaning of SES for the accessibility and utilisation of biologic IBD treatment is of interest under consideration of the relevance of the timing of the disease management. There is limited evidence that low SES may be a disadvantage in accessing biologic treatment, and details on this are largely unknown [7].

### Objectives/hypotheses

The study aims to investigate whether income, education, and occupation are associated with time to treatment onset from diagnosis of UC or CD in Danish patients. Based on evidence from previous investigations, a positive association between SES and the timely start of biologic treatment seems plausible, assuming that higher SES is related to patients’ engagement and rational behaviour in pursuing the improvement of their condition [8].

## Methods

### Design and population

**Fig. 1:**
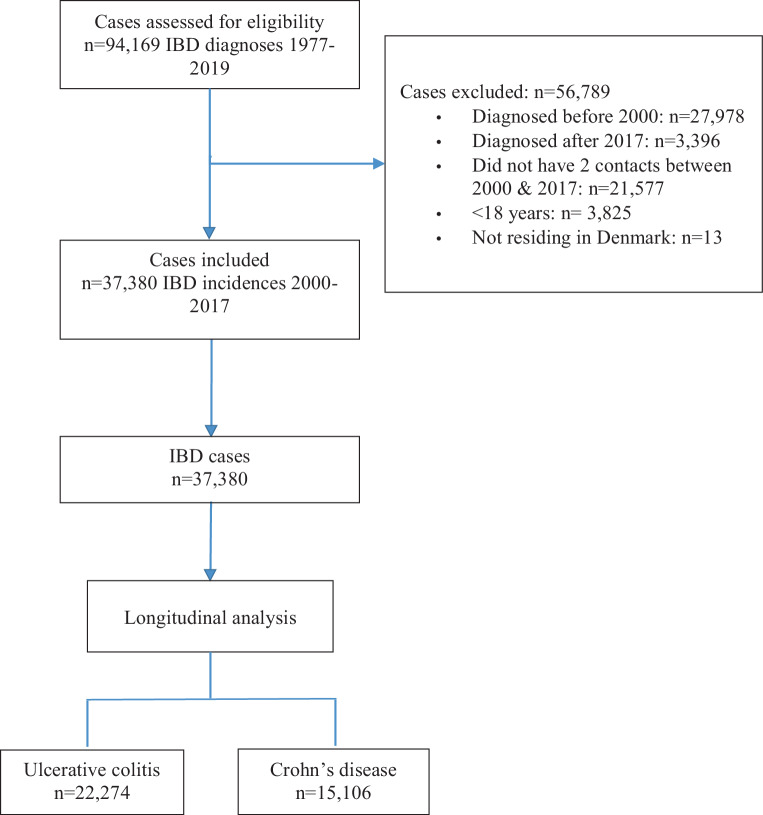
Flow chart of the patient population inclusion process

We conducted an observational population-based cohort study using Danish nationwide register data. All IBD (CD or UC) incidences in the Danish adult population with an initial hospital contact between January 1, 2000, and December 31, 2017 were included. The diagnosis (index) date was the day of the first hospital contact after 2000 with a diagnostic code for IBD. According to our inclusion criteria, incident cases were patients with at least two hospital contacts with IBD diagnosis (ICD-10 K50 & K51) within five years at a Danish hospital. To ensure the correct identification of new incidences, we checked the register for IBD prevalence in the period 1995–1999. Fig. 1 depicts the inclusion process of the studied patients.

Cases were followed up until the onset of biologic treatment. Patients without any registration of biologic treatment were censored at the date of IBD-related surgery (procedure codes starting with “KJFK”/“KJFB”/“KJFA58”/“KJFA38”/“KJFA6”/“KJFH”/“KJFF”), emigration, death, or at the end of the observation period (31/12/2018).

The study was carried out in accordance with the Strengthening the Reporting of Observational Studies in Epidemiology (STROBE) guidelines [9].

### Danish national registers

Each official resident in Denmark is assigned a unique personal identification number, which in its encrypted form is used to link person data from different registers. The Danish national population registers are of high quality and comprehensiveness and offer the opportunity to study complete population samples using systematically collected long-term data [10]. Demographic patient information was retrieved from the Civil Registration System [11]. Data on income, education and occupation were retrieved from the Income Statistics Register [12], the Student Register [13] and the Employment Classification Module [14], respectively. The National Patient Register (NPR) provided medical data on comorbidity, disease management activities and hospital contacts [15].

### Variables

#### Onset of biologic treatment

The main outcome is indicated as the number of days between initial IBD diagnosis and the date of the first received dose of a Tumor Necrosis Factor‑α inhibitor (TNF-i) (treatment code: BOHJ18A1/BOHJ18A3/BOHJ18A4), an anti-integrin (BOHJ19H4) or anti-interleukin (BOHJ18B3) as recorded in the NPR.

#### Socioeconomic status

The socioeconomic indicators included measures of education, income and occupation. They were handled as separate characteristics to explore each factor’s individual association with the outcome.

**Educational level: **The highest completed education before the index date was classified into four categories (lower secondary education or less, upper secondary education, vocational education and academic education) based on the 2011 International Standard Classification of Education [16].

**Equivalent disposable household income: **Baseline income was specified by the annual disposable amount of money available to the subjects based on the total household income relative to the number of persons living in the respective household. Data from the year preceding the index date was used toallocate the patients. Thereby, we made sure that the IBD diagnosis and disease course would not interact with the income subsequently. Baseline income was divided into quartiles.

**Occupational status: **Occupational status in the year preceding the IBD diagnosis as according to the Employment Classification Module described the main source of income in six categories: Employed/self-employed, unemployed including receivers of governmental financial support (social aid), student, retired, sick leave and other in case the main income source was registered as not identifiable.

#### Covariates

The analyses were adjusted for age, sex, presence of comorbidities and index year, as the availability of biologic drugs increased substantially after 2004. Prevalence of comorbidity was identified via ICD-10 codes as registered during hospital contact. Diseases considered most relevant in the uptake of biologic treatment were included. These comprised hypertension, cerebrovascular disease, psychiatric disorders, rheumatoid arthritis, other inflammatory diseases (psoriasis, multiple sclerosis, ankylosing spondylitis), tuberculosis and hepatitis B/C [17–19]. Comorbidities leading to hospital contact within one year before the IBD diagnosis were considered clinically relevant for the analysis.

### Statistical analysis

Cox proportional hazards regression models were applied to analyse the association between SES and first biologic treatment. We estimated hazard ratios (HRs) with corresponding 95% confidence intervals (CIs) and a significance level of α = 5%. The models were grouped by IBD type to map disease type-specific associations in the analyses.

The proportional hazards assumption was assessed graphically using Schoenfeld residuals. Consequently, income was included with a time-varying coefficient. Martingale residuals were used to evaluate the model fit regarding the linearity of covariates. We did not adjust the *p*-values for multiple testing, as each analysis was conducted individually. The E‑value was computed for estimates as a sensitivity analysis of the robustness of the models where the 95%-CI excluded the null [20]. Missing data due to unavailable information from registers were few (< 3%) and mean imputation was conducted. All analyses were conducted in Stata version 17.

## Results

### Population characteristics

There were 37,380 IBD cases that met the inclusion criteria of whom 15,106 (40.4%) and 22,274 (59.6%) were CD and UC cases, respectively. The median age of patients with CD and UC was 40.0 and 47.0 years and a larger share of the patients was female (55.8% for CD and 51.7% for UC). The largest part of the population had a vocational education with 40.0% in the CD and 42.5% in the UC group. Annual disposable income in the patient population with CD was 23,966 and 25,574 € in the population with UC. A total of 54.4% of patients with CD and 56.8% of the patients with UC were employed at the time of their IBD diagnosis whilst 1.6% of patients with CD and 1.4% among patients with UC were on sick leave. Retirement was more prevalent in the UC than in the CD group (27.1% vs. 23.0%). In both patient groups, a total of 6.8% presented comorbidities of which the most frequent was hypertension (57.1 and 64.8% in CD and UC, respectively). Demographic, socioeconomic and comorbidity characteristics at the index date are summarised in Table 1.

**Table 1:** Baseline characteristics of Danish patients with CD and UC diagnosed between 2000 and 2017

Factor	Level/Unit	CD (*n* = 15,106)	UC (*n* = 22,274)
Age at diagnosis, median (IQR)	Years	40.0 (27.0; 57.0)	47.0 (33.0; 62.0)
Sex	Female	8427 (55.8%)	11,505 (51.7%)
Educational level^a^	Lower secondary or less	4850 (32.1%)	6155 (27.6%)
	Upper secondary	1555 (10.3%)	1888 (8.5%)
	Vocational	6044 (40.0%)	9462 (42.5%)
	Academic	2657 (17.6%)	4769 (21.4%)
Equivalent annual income, median (IQR)^b^	Euro (€)	23,966 (17,776; 31,498)	25,574 (18,855; 33,833)
Occupation	Employed	8222 (54.4%)	12,653 (56.8%)
	Unemployed/social aid	866 (5.7%)	858 (3.9%)
	Student	1832 (12.1%)	1927 (8.7%)
	Retired	3479 (23.0%)	6028 (27.1%)
	Sick leave	243 (1.6%)	305 (1.4%)
	Other^c^	464 (3.1%)	503 (2.3%)
Comorbidity	Total	1020 (6.8%)	1524 (6.8%)
	Hypertension	582 (57.1%)	987 (64.8%)
	Cerebrovascular disease	160 (15.7%)	299 (19.6%)
	Rheumatoid arthritis	79 (7.7%)	112 (7.3%)
	Psychiatric disorders	193 (18.9%)	236 (15.5%)
	Other inflammatory diseases^d^	102 (10.0%)	86 (5.6%)
	Tuberculosis	11 (1.1%)	10 (0.7%)
	Hepatitis B/C	11 (1.1%)	5 (0.3%)

Missing values: ^a^989 (2.6%); ^b^262 (< 0.1%)

^c^no identifiable dominant income source

^d^psoriasis, multiple sclerosis, ankylosing spondylitis

### Patient follow-up

By the end of the observation period, 4346 (28.8%) patients with CD and 2902 (13.0%) patients with UC had received biologic treatment. The median follow-up time from index date to either start of biologic treatment, end of observation on December, 31st 2017 or earlier censoring was 5.9 years (6.7 years for UC and 4.5 years for CD).

### Start of biologic treatment

Hazard ratios for the respective SES categories are presented in Table 2. Adjusted for age, sex, comorbidity and diagnosis year, neither the CD nor the UC population’s educational background was associated with time to treatment initiation. Patients with CD in the two highest income quartiles showed a higher rate of treatment initiation than patients in quartile 1 (HR 1.16; 95% CI: 1.04; 1.30 and HR 1.15; 95% CI: 1.03; 1.30). This difference was not found in patients with UC. Patients with UC and “other” occupational status received biologic treatment earlier than the employed population (HR 1.36; 95% CI: 1.11; 1.66). There was no evidence for an overall association of employment status and time to treatment start.

**Table 2:** Analysis results for hazard ratios of receiving biologic treatment by SES

	Patients with CD		Patients with UC	
Variable/Level	HR (95% CI)^a^	*p*-value	HR (95% CI)^a^	*p*-value
*Educational level*				
Lower secondary	1 (reference)	0.693	1 (reference)	0.165
Upper secondary	1.00 (0.91; 1.10)		1.10 (0.97; 1.24)	
Vocational	1.03 (0.96; 1.11)		0.98 (0.89; 1.07)	
Academic	0.99 (0.90; 1.08)		0.95 (0.85; 1.06)	
*Income*				
Q1 (< 18,448 €)	1 (reference)	0.017	1 (reference)	0.406
Q2 (18,448–24,939 €)	1.03 (0.92; 1.15)		1.00 (0.86; 1.17)	
Q3 (24,939–32,946 €)	1.16 (1.04; 1.30)		1.11 (0.96; 1.28)	
Q4 (> 32,946 €)	1.15 (1.03; 1.30)		1.07 (0.92; 1.23)	
*Occupational status*				
Employed	1 (reference)	0.283	1 (reference)	0.062
Unemployed/social aid	0.98 (0.87; 1.06)		0.95 (0.79; 1.14)	
Student	0.98 (0.89; 1.06)		1.06 (0.95; 1.19)	
Retired	0.94 (0.83; 1.06)		0.97 (0.83; 1.12)	
Sick leave	0.80 (0.63; 1.02)		1.11 (0.84; 1.45)	
Other	0.88 (0.75; 1.04)		1.36 (1.11; 1.66)	

^a^adjusted for age, sex, comorbidities and calendar year of diagnosis

E‑values for the lower 95% CI were computed at 1.20 and 1.17 for income quartiles 3 and 4 in the CD group and 1.36 for “other” occupational status in patients with UC (supplementary tables S1 and S2).

## Discussion

To our knowledge, this is the first study to investigate the association between SES and time to biologic treatment onset in Denmark. The results did not support the hypothesis that there is unequal disease management between patients with IBD of different educational levels, incomes, or occupational statuses. Despite education- and income-related differences in care seeking behaviour [6, 21], it could not be confirmed that socioeconomic subgroups experience a delay in treatment onset compared with their reference groups. Time to treatment was largely equal with a slight tendency of higher income in patients with CD being related to receiving biologic treatment earlier. Cost-related motives in patients seem rather implausible under fully state-covered treatment expenses. Furthermore, the association is not highly robust against unmeasured confounding as reflected by the E‑values, nor does it hold in the UC group. Our study shows few distinct inequalities in time to treatment, though they cannot be interpreted as systematic, as the overall results show no or weak associations that vary between IBD types.

Few studies have investigated treatment adherence and found socioeconomic differences in patient behaviour to be heterogeneous: Results ranged from positive correlations [22] over no difference [23] to negative correlations between SES and adherence [24]. These multi-directional results suggest that utilisation patterns in IBD therapy vary more between nations than between individual conditions. The authors of a review [25] underline that a country’s health system is likely the most impactful factor for socioeconomic inequalities.

Bernstein et al. [7] conducted a methodologically and thematically similar study on a Canadian cohort of patients with IBD. The authors investigated the association between various healthcare outcomes and individual or area-level socioeconomic factors. Although their results on access to biologic treatment were not fully consistent, they found an overall negative association between economic deprivation, being a receiver of financial aid and social services, and chances to receive biologic treatment. The presented results are only partly consistent with Scandinavian studies that investigated SES-related inequities in healthcare utilisation for other conditions. Gundgaaard [26] found that income-related inequality in healthcare utilisation is affected by the degree of co-payment patients are required to make. This is not transferable to biologic treatment administered at Danish public hospitals. A recent Danish register study named the site of care, but not individual SES, to be of relevance for early biologic treatment start [27]. The present study results were consistent with findings from Nahon et al. [23]. who reported absence of an association between socioeconomic deprivation or educational level and utilisation of biologic treatment by French patients with IBD.

The partial discrepancy between this study and other studies demonstrating inequality may be caused by differences in clinical settings and between populations [28]. In Denmark, the initiation of biologic IBD treatment is administered only in the public hospital sector. Its clinical necessity is assessed according to practitioners’ and patients’ opinion [29]. Varying approaches to disease management limit the generalisability to other contexts.

There are several strengths to this study. Firstly, this study covered all Danish adult IBD incidences between 2000 and 2017 as to the defined inclusion criteria, ensuring high validity and power of the findings. Secondly, the use of national register data formed a comprehensive and objective source with large coverage and high quality. Thirdly, the requirement of two hospital contacts with IBD diagnosis reduced the risk of misdiagnoses [30]. Fourthly, the separate exposure indicators rather than composite indexes made it possible to identify potential associations related to distinct factors. Furthermore, the historic longitudinal study design endorsed the inclusion of pre-diagnostic patient characteristics in contrast to previous research that focused on socioeconomic consequences of IBD diagnoses.

The major limitations of the study include the following: The register data did not allow to take clinical patient characteristics into consideration that might be connected to medical decisions. Register data are restricted to a certain set of variables to be investigated. Individual and subjective factors in disease management like the degree of disability and patient involvement could therefore not be included. In light of missing clinical information, the research was based on the assumption of equal needs, i.e., that there was no difference in treatment needs between the studied patient groups, which, however, could not be verified. The sensitivity analysis indicated rather weak robustness of the results. This emphasises the need to account for additional relevant clinical information (e.g., disease severity) that is tightly connected to treatment decisions. We therefore advise that disease characteristics be considered to account for treatment needs and medical prognoses. Moreover, the low robustness of the models could explain the limited comparability to other studies that point towards socioeconomic treatment inequality.

In conclusion, administration of biologic treatment in the investigated IBD patient population was found to be largely equitable across education, income, and occupational groups. The findings from the present study could suggest that the specific national characteristics in IBD management outweigh the variabilities mentioned by other studies. Seen in combination with investigations of health service use in other domains, it is suggested that hospital-centered IBD-care with tax-funded treatment delivery is equal in distribution and that the previously reported discrepancies may have been caused by a higher degree of heterogeneity in other patient populations and organisational dissimilarities to the Danish approach.

To understand better the mechanisms behind SES-related health inequity in patients with IBD, it is necessary to complement future register research with clinical information, patient-informed survey data and qualitative input on decision-making processes from healthcare providers.

## Supplementary Information

Results from the sensitivity analyses

## Data Availability

Supporting data is not available due to legal restrictions in connection with Statistics Denmark’s confidentiality politics.
