# Supplementary material for: Socioeconomic equality in initiation of biologic treatment in Danish patients with inflammatory bowel disease
Source: Wien Klin Wochenschr. 2024 May 24;137(15-16):463–9. doi: 10.1007/s00508-024-02376-8 (PMC12370555; doi:10.1007/s00508-024-02376-8)
Supplement: Supplementary file 1 — Results from the sensitivity analyses [file 508_2024_2376_MOESM1_ESM.docx]

| *Supplementary Table S1: Sensitivity analysis of the results - CD patients* | | | | | | |
| --- | --- | --- | --- | --- | --- | --- |
| **Variable/ Level** | **HR** | **E-value HR** | **CI (lower limit)** | **E-value CI (lower limit)** | **CI (upper limit)** | **E-value CI (upper limit)** |
| **Educational level** |  |  |  |  |  |  |
| Lower secondary | 1.00 | . | . | . | . | . |
| Upper secondary | 1.00 | 1.00 | 0.91 | . | 1.10 | . |
| Vocational | 1.03 | 1.17 | 0.96 | . | 1.11 | . |
| Academic | 0.99 | 1.09 | 0.90 | . | 1.08 | . |
| **Income** |  |  |  |  |  |  |
| Q1 | 1.00 | . | . | . | . | . |
| Q2 | 1.03 | 1.17 | 0.92 | . | 1.15 | . |
| Q3 | 1.16 | 1.45 | 1.04 | 1.20 | 1.30 | . |
| Q4 | 1.15 | 1.44 | 1.03 | 1.17 | 1.30 | . |
| **Occupational status** |  |  |  |  |  |  |
| Employed | 1.00 | . | . | . | . | . |
| unemployed/social aid | 0.98 | 1.13 | 0.87 | . | 1.06 | . |
| student | 0.98 | 1.13 | 0.89 | . | 1.06 | . |
| retired | 0.94 | 1.26 | 0.83 | . | 1.06 | . |
| sick leave | 0.80 | 1.61 | 0.63 | . | 1.02 | . |
| other | 0.88 | 1.41 | 0.75 | . | 1.04 | . |

| *Supplementary Table S2: Sensitivity analysis of results - UC patients* | | | | | | |
| --- | --- | --- | --- | --- | --- | --- |
| **Variable/ Level** | **HR** | **E-value** | **CI (lower limit)** | **E-value CI (lower limit)** | **CI (upper limit)** | **E-value CI (upper limit)** |
| **Educational level** |  |  |  |  |  |  |
| Lower secondary |  | . | . | . | . | . |
| Upper secondary | 1.10 | 1.34 | 0.97 | . | 1.24 | . |
| Vocational | 0.98 | 1.13 | 0.89 | . | 1.07 | . |
| Academic | 0.95 | 1.23 | 0.85 | . | 1.06 | . |
| **Income** |  |  |  |  |  |  |
| Q1 |  | . | . | . | . | . |
| Q2 | 1.00 | 1.00 | 0.86 | . | 1.17 | . |
| Q3 | 1.11 | 1.36 | 0.96 | . | 1.28 | . |
| Q4 | 1.07 | 1.07 | 0.92 | . | 1.23 | . |
| **Occupational status** |  |  |  |  |  |  |
| Employed |  | . | . | . | . | . |
| unemployed/social aid | 0.95 | 1.23 | 0.79 | . | 1.14 | . |
| student | 1.06 | 1.25 | 0.95 | . | 1.19 | . |
| retired | 0.97 | 1.17 | 0.83 | . | 1.12 | . |
| sick leave | 1.11 | 1.36 | 0.84 | . | 1.45 | . |
| other | 1.36 | 1.78 | 1.11 | 1.36 | 1.66 | . |
